# Comparison of cardiovascular risk profiles of patients with type A aortic dissection and thoracic aortic aneurysm: a retrospective multicentre study

**DOI:** 10.1136/bmjopen-2024-097306

**Published:** 2025-09-23

**Authors:** Aytug U Tirpan, Onur Baris Dolmaci, Evert K Jansen, Jos W R Twisk, Robert J M Klautz, Nimrat Grewal

**Affiliations:** 1 Department of Cardiothoracic Surgery, Amsterdam UMC Location AMC, Amsterdam, The Netherlands; 2 Department of Cardiology, Onze Lieve Vrouwe Gasthuis, Amsterdam, The Netherlands; 3 Department of Epidemiology and Data Science, Amsterdam University Medical Centres, Amsterdam, The Netherlands; 4 Department of Cardiothoracic Surgery, Leiden University Medical Center, Leiden, The Netherlands; 5 Department of Anatomy and Embryology, Leiden University Medical Center, Leiden, The Netherlands; 6 Department of Cardiothoracic Surgery, Yale New Haven Hospital, Yale University School of Medicine, New Haven, Connecticut, USA

**Keywords:** Risk Factors, Cardiovascular Disease, Cardiothoracic surgery, Risk Assessment, CARDIOLOGY

## Abstract

**Abstract:**

**Objective:**

A thoracic aortic aneurysm (TAA) is often considered a precursor to an acute type A aortic dissection (ATAAD), a life-threatening condition requiring immediate surgical intervention. While both conditions share histopathological similarities, less is known about their overlap in clinical cardiovascular risk factors. This study aimed to map the cardiovascular disease burden in patients with ATAAD and compare it with patients with TAA.

**Design:**

A multicentre retrospective study.

**Setting:**

The data were collected from electronic health records of two academic hospitals located in the Netherlands.

**Participants:**

Patients who were treated surgically for ATAAD or TAA between 2000 and 2022 were eligible. This study included 731 patients with ATAAD and 480 patients with TAA.

**Results:**

Hypertension was equally prevalent in both groups (50.9% vs 50.6%, p=0.921). Diabetes was uncommon (3.3% vs 6.7%, p=0.638). Hyperlipidaemia (9.6% vs 20.0%, p=0.001) and peripheral arterial disease (8.8% vs 22.7%, p<0.001) were less prevalent in patients with ATAAD. Smoking was more prevalent in patients with ATAAD (35.9% vs 33.2%, p=0.014).

**Conclusion:**

This study suggests distinct cardiovascular risk profiles in patients with ATAAD and patients with TAA, highlighting the importance of tailored treatment strategies for aortic disease. Further research is needed to investigate the pathophysiological mechanisms underlying these differences and their impact on thoracic aortopathy.

**Strengths and limitations of this study**

- A multicentre study with a large number of included patients.
- Including patients from 2000 to 2020 with a retrospective design.
- Multiple imputation was used, which reduces bias and maximises use of available information.

## Introduction

 An acute type A aortic dissection (ATAAD) is defined as a tear in the intimal layer of the aortic wall proximal to the left subclavian artery, enabling blood to surge between the tunica intima and media, ‘dissecting’ the wall layers into a true and ‘false’ lumen.^1^ An ATAAD is a life-threatening condition with an incidence of 5.93 cases per 100 000 person-years.^2^ Untreated dissections are highly lethal with a mortality rate up to 84%,^3^ due to the occurrence of an aortic rupture, a cardiac tamponade, myocardial ischaemia and/or a cerebrovascular accident.^1 4^ After emergency surgical treatment, a 30-day mortality rate of 17%–20% is reported.^5^

The exact pathogenesis of an ATAAD has not yet been elucidated. A risk factor for the development of an ATAAD is a pathological aortic widening, called a thoracic aortic aneurysm (TAA).^6 7^ TAAs are a complex entity, which can broadly be classified as syndromic, non-syndromic and sporadic. Interestingly, in previous work, we showed that all cases of TAAs—independent of the underlying cause—are unitedly characterised by similar clinical, pathological and early embryonic maturation defects. Specifically, these defects include incomplete differentiation of vascular smooth muscle cells, leading to an imbalance between contractile and synthetic cells in the medial layer. This is accompanied by extracellular matrix accumulation and a significantly thin intimal layer, which weakens the structural integrity of the aortic wall. Clinically, this shared vulnerability suggests that histopathological characteristics may help refine patient risk stratification beyond the current reliance on aortic diameter alone.^8^,^13^

Research has shown that patients with ATAAD exhibit similar histopathological features as patients with TAA. These histopathological characteristics, such as extracellular matrix accumulation and reduced contractile smooth muscle cells, are not only present in dilated aortas of patients with TAA but are also found in the aortas of patients with ATAAD without obvious dilation. This suggests an underlying structural vulnerability in the aortic wall that is common to both conditions, which could be clinically relevant for identifying high-risk patients beyond geometric criteria.^8^,^1113^

As mentioned above, the pathogenesis appears to overlap in several histopathological aspects. However, the aortic diameter is currently the primary decisive marker to consider patients for elective prophylactic aortic replacement surgery. Although clinical risk stratification is currently solely based on a geometric criterion, several studies have already shown that this threshold is an insufficient predictor of ATAAD development,^14^,^17^ as 87% of acute aortic events occur at a diameter below 45 mm.^14^ These clinical and fundamental studies thus provide evidence that there is a need for a more patient-tailored risk stratification in thoracic aortopathy. Despite vigorous research efforts in past years, no strong clinical or biological predictive marker has been established to identify patients at risk for an acute aortic event. Therefore, we sought to determine potential clinical risk factors for ATAAD other than the aortic diameter. In this study, we examine the cardiovascular risk factors of patients with ATAAD at the time of diagnosis and subsequently compare them with those of patients with TAA.

## Methods

### Settings and population

A multicentre retrospective study was conducted in two academic hospitals in the Netherlands: Amsterdam University Medical Center and Leiden University Medical Center. The surgical databases were searched to identify patients (≥18 years) who underwent an aortic replacement between 1 January 2000 and 31 December 2022. Patients who required surgical replacement of the aortic root, ascending aorta or aortic arch due to an aortic dissection were included in the ATAAD group. Patients with an aortic diameter of ≥45 mm without evidence of dissection were included in the TAA group. Surgical decisions were made in accordance with the European Society of Cardiology (ESC) and Society of Thoracic Surgeons (STS)/European Association for Cardio-Thoracic Surgery (EACTS) guidelines.^18 19^ Although the general threshold for elective surgery for TAA is ≥55 mm, the guidelines also state that surgery with smaller diameters may be considered in specific situations. Analogous to the ESC and STS/EACTS guidelines, surgery below 55 mm was performed in patients with connective tissue disorders (eg, Marfan syndrome), risk factors (eg, hypertension, aortic growth rate >3 mm/year or family history) or concomitant aortic valve/root pathology. All surgical indications were determined by multidisciplinary heart teams, ensuring guideline-based decision-making.

**Figure 1:**
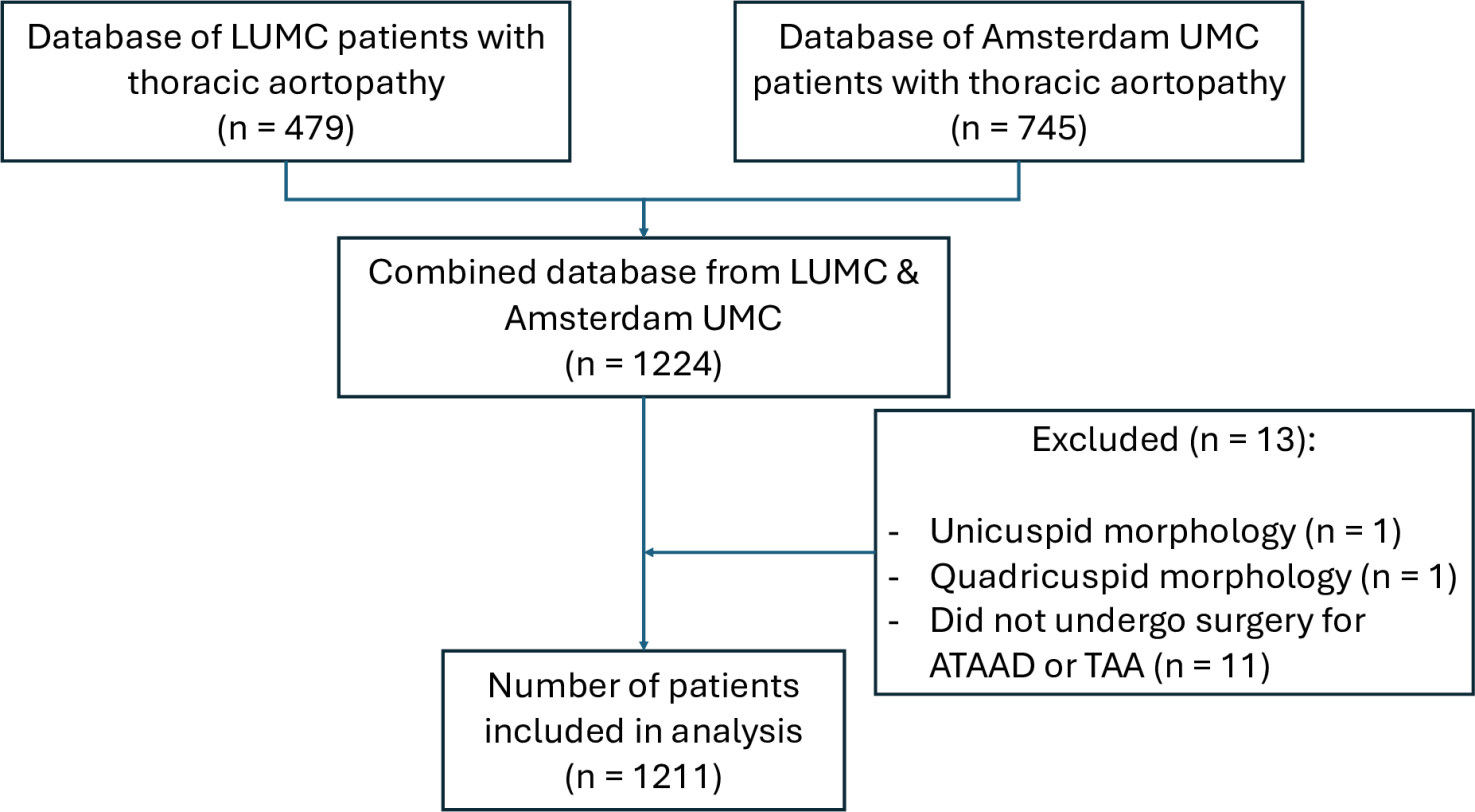
Flow chart summarising the data collection process. ATAAD, acute type A aortic dissection; LUMC, Leiden University Medical Center; TAA, thoracic aortic aneurysm; Amsterdam UMC, Amsterdam University Medical Center.

To ensure a targeted and homogeneous study population, thoraco-abdominal aneurysms were excluded from the analysis. Thoracic false aneurysms were included only when associated with a spontaneous ATAAD, excluding cases due to trauma or iatrogenic causes. In addition, TAAs arising from chronic dissections and cases with unicuspid and quadricuspid aortic valves were excluded due to their rarity (figure 1).

### Data collection

The electronic patient records were searched to obtain baseline characteristics, cardiovascular risk factors, imaging and surgical characteristics.

Patients were scored as hypertensive if they had a mean systolic blood pressure of 140 mm Hg or greater, and/or a diastolic blood pressure of 90 mm Hg or greater on three separate occasions, were taking antihypertensive drugs or when hypertension was reported in the patients’ medical history.^20^ Diabetes mellitus was scored if patients were taking blood sugar lowering medication or when documented in the medical history, had a fasted blood sugar level of ≥7 mmol/L or had a blood sugar level of ≥11.1 mmol/L.^20^ Hyperlipidaemia was scored if patients had a total cholesterol of ≥5 mmol/L, were using lipid-lowering medication or if the condition was reported in the patients’ medical history.^20^ Patients were scored as smokers if they had ever smoked in the past or were current smokers. Peripheral arterial disease was scored if documented in the patient’s medical history, which is based on ESC guidelines: a resting ankle-brachial index ≤0.90. If >0.90, a >20% post-exercise decrease and/or supportive imaging findings also support the diagnosis.^19^

The aortic valve morphology was primarily assessed by studying surgical reports and alternatively using transthoracic/transoesophageal ultrasound, or CT. The aortic diameter was primarily measured on CT scans at the level of the ascending aorta, and otherwise transthoracic or transoesophageal ultrasound was used to measure the diameter. Genetic syndromes (eg, Marfan syndrome, Ehlers-Danlos syndrome and Loeys-Dietz syndrome) were diagnosed through DNA testing by the clinical geneticist and/or established by a physician as a clinical diagnosis. Patients exhibiting genetic heterogeneity of a TAA were categorised as having familial thoracic aortic aneurysm and dissection.^21^

### Data analysis

Continuous variables are presented as mean and SD, while dichotomous variables are presented as frequencies and percentages. Normality and skewness were assessed visually for all variables. Missing data were imputed using multiple imputation by fully conditional specification (chained equations) with predictive mean matching. 40 imputed datasets were generated, including all variables in the analysis model. A binary logistic regression analysis was performed on each of the imputed datasets, and the results were pooled to obtain overall ORs and 95% CIs. A univariable approach was first used to calculate the ORs. Following this, a multivariable approach was employed that incorporated all variables. In additional analyses, demographic differences were compared between patients with ATAAD <45 mm and those with TAA, and between patients with ATAAD ≥45 mm and those with TAA. Significance was defined as p<0.05. All statistical analyses were conducted using IBM SPSS for Windows V.28.0.

### Patient and public involvement statement

Patients and/or the public were not involved in the design, or conduct, or reporting or dissemination plans of this research.

## Results

### Baseline characteristics

This study included a total of 1211 patients, including 731 patients with ATAAD (61.4% male) and 480 patients with TAA (68.5% male). The mean age among patients with ATAAD was 62.1 ± 12.6 years, while patients with TAA had an average age of 62.9 ± 11.4 years. All baseline and surgical preoperative characteristics are shown in table 1.

**Table 1:** Characteristics and prevalence of cardiovascular risk factors in patients with ATAAD and patients with TAA, a univariable analysis

Characteristic	ATAAD, n=731	TAA, n=480	OR (95% CI)	P value
Sex			0.731 (0.573 to 0.932)	**0.012**
Male	449 (n=449) (61.4)	329 (n=329) (68.5)		
Female	282 (n=282) (38.6)	151 (n=151) (31.5)		
Age (years)	62.1±12.6 (n=731)	62.9±11.4 (n=477)	0.995 (0.985 to 1.004)	0.287
Height (cm)	176.5±10.3 (n=687)	176.2±10.6 (n=425)	1.006 (0.995 to 1.018)	0.272
Weight (kg)	82.4±16.0 (n=701)	83.1±16.4 (n=425)	0.999 (0.991 to 1.006)	0.727
Genetic syndrome*	45 (n=730) (6.2)	12 (n=247) (4.9)	1.098 (0.462 to 2.609)	0.830
Aortic valve morphology			17.187 (11.780 to 25.076)	**<0.001**
Bicuspid aortic valve	39 (n=724) (5.4)	203 (n=404) (50.2)		
Tricuspid aortic valve	685 (n=724) (94.6)	201 (n=404) (49.8)		
Hypertension	372 (n=731) (50.9)	243 (n=480) (50.6)	1.011 (0.803 to 1.272)	0.928
Diabetes mellitus	24 (n=731) (3.3)	32 (n=480) (6.7)	0.475 (0.276 to 0.817)	**0.007**
Hyperlipidaemia	70 (n=731) (9.6)	96 (n=480) (20.0)	0.424 (0.304 to 0.591)	**<0.001**
Smoking	239 (n=666) (35.9)	157 (n=473) (33.2)	1.129 (0.880 to 1.448)	0.340
Peripheral arterial disease	64 (n=731) (8.8)	67 (n=295) (22.7)	0.287 (0.195 to 0.421)	**<0.001**
COPD	63 (n=731) (8.6)	25 (n=295) (8.5)	0.839 (0.504 to 1.397)	0.498
Creatinine (μmol/L)	84.7±37.5 (n=705)	88.1±25.2 (n=478)	0.997 (0.993 to 1.000)	0.091
Aortic diameter (mm)*	44.8±11.8 (n=662)	52.2±6.6 (n=187)	0.933 (0.915 to 0.950)	**<0.001**

Categorical variables are presented as n (n=initially available cases) (%) and continuous variables as mean±SD (n=initially available cases).

P-values <0.05 are depicted in bold.

* More detailed information can be found in table 3.

ATAAD, acute type A aortic dissection; COPD, chronic obstructive pulmonary disease; TAA, thoracic aortic aneurysm.

### Cardiovascular risk factors

**Figure 2:**
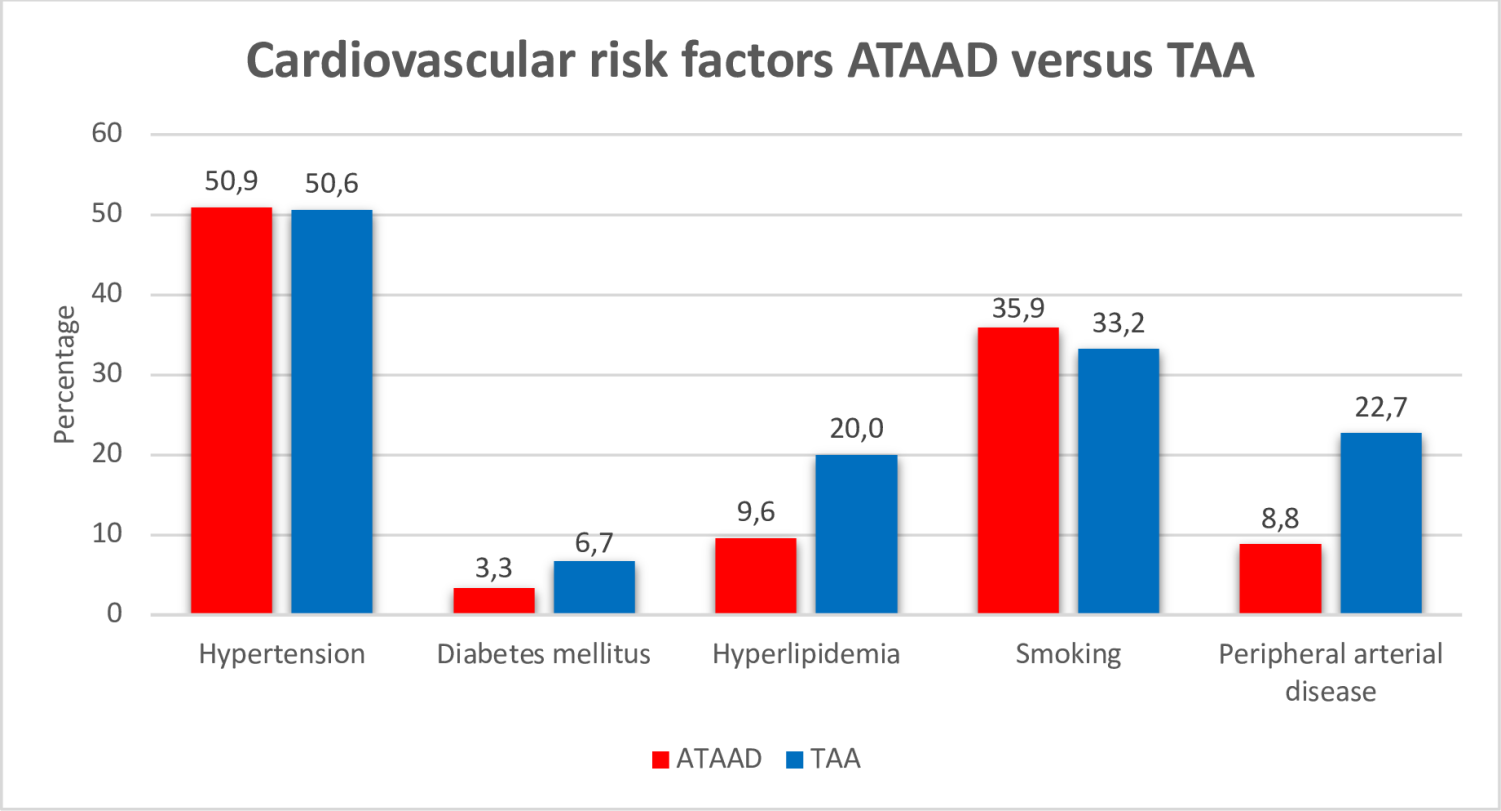
Prevalence of cardiovascular risk factors in patients with ATAAD versus patients with TAA. Data are presented as n (%). ATAAD, acute type A aortic dissection; TAA, thoracic aortic aneurysm.

The prevalence of cardiovascular risk factors is presented for both groups in figure 2. Patients with ATAAD had a similar prevalence of hypertension compared with patients with TAA (50.9% vs 50.6%, respectively, p=0.921). The prevalence of diabetes mellitus was also similar between the two groups (3.3% vs 6.7%, respectively, p=0.638). Hyperlipidaemia (9.6% vs 20.0%, respectively, p=0.001) and peripheral arterial disease (8.8% vs 22.7%, respectively, p<0.001) were less common in ATAAD, whereas smoking was more frequent (35.9% vs 33.2%, respectively, p=0.014).

### Risk factors in the multivariable analysis

In the multivariable analysis, sex, aortic valve morphology, hyperlipidaemia, peripheral arterial disease and aortic diameter remained significant. Diabetes mellitus became non-significant, while smoking and height became significant (table 2).

**Table 2:** Results of multivariable analysis of patient characteristics and cardiovascular risk factors in patients with ATAAD versus patients with TAA

	OR	% 95 CI	P value
Sex	0.568	0.370 to 0.873	**0.010**
Age	0.985	0.970 to 1.000	0.051
Height	1.047	1.023 to 1.071	**<0.001**
Weight	0.997	0.985 to 1.009	0.639
Genetic syndrome*	0.671	0.283 to 1.590	0.363
Tricuspid aortic valve	20.404	13.163 to 31.627	**<0.001**
Hypertension	1.016	0.737 to 1.401	0.921
Diabetes mellitus	0.827	0.375 to 1.825	0.638
Hyperlipidaemia	0.473	0.302 to 0.740	**0.001**
Smoking	1.561	1.095 to 2.225	**0.014**
Peripheral arterial disease	0.304	0.192 to 0.482	**<0.001**
COPD	0.668	0.366 to 1.219	0.188
Creatinine	0.997	0.992 to 1.001	0.136
Aortic diameter	0.932	0.913 to 0.952	**<0.001**

P-values <0.05 are depicted in bold.

* Marfan syndrome, Ehlers-Danlos syndrome and Loeys-Dietz syndrome.

ATAAD, acute type A aortic dissection; COPD, chronic obstructive pulmonary disease; TAA, thoracic aortic aneurysm.

### Aortic diameters

The analyses of the aortic diameter of all patients and the prevalence of genetic syndromes are shown in table 3. Overall, there was no significant difference in prevalence of genetic syndromes between patients with ATAAD and patients with TAA.

**Table 3:** Frequencies of the aortic diameter and genetic syndromes in patients with ATAAD and patients with TAA

Characteristic	ATAAD, n=731	TAA, n=480
Aortic diameter		
<40 mm	189 (28.5)	0 (0.0)
40–44 mm	126 (19.0)	0 (0.0)
45–49 mm	133 (20.1)	70 (37.4)
50–54 mm	105 (15.9)	58 (31.0)
≥55 mm	109 (16.5)	59 (31.6)
Genetic syndrome*		
Marfan	38 (5.2)	10 (4.0)
Ehlers-Danlos	4 (0.5)	0 (0.0)
Loeys-Dietz	4 (0.5)	1 (0.4)
FTAAD	13 (1.8)	2 (0.8)
Other†	9 (1.2)	3 (1.2)

Data are presented as n (%).

* Not all data were available for patients with TAA.

† Different types of genetic mutations associated with TAAs or dissections.

ATAAD, acute type A aortic dissection; FTAAD, familial thoracic aortic aneurysm and dissection; TAA, thoracic aortic aneurysm.

According to the current European and American guidelines, the threshold for aortic replacement surgery is 55 mm and greater.^18 21^ Notably, 83.5% of the patients with ATAAD had an aortic diameter below this threshold. Interestingly, 47.7% of the patients in the ATAAD group had an aortic diameter below 45 mm, which is the lowest threshold for aortic replacement surgery according to the guidelines.

Therefore, additional comparisons were made between the baseline characteristics and cardiovascular risk factors of patients with ATAAD with an aortic diameter below 45 mm or ≥45 mm, which were subsequently compared with patients with TAA (table 4). Of all cardiovascular risk factors, only diabetes mellitus was more common in patients with ATAAD with an aortic diameter below 45 mm compared with those with an aortic diameter of ≥45 mm. ATAAD patients with diabetes and a diameter of ≥45 mm were further compared with patients with TAA, and diabetes was less common in patients with ATAAD (online supplemental table 1).

**Table 4:** Characteristics and prevalence of cardiovascular risk factors in patients with ATAAD with a diameter of <45 mm or ≥45 mm, versus patients with TAA

Characteristic	ATAAD <45 mm, n=315	ATAAD ≥45 mm, n=347	TAA ≥45 mm, n=187
Sex			
Male	189 (60.0)	211 (60.8)	329 (68.5)
Female	126 (40.0)	136 (39.2)	151 (31.5)
Age	61.2±12.7	62.3±12.9	62.9±11.4
Height	175.6±10.0	177.1±10.4	176.2±10.6
Weight	81.1±16.3	83.0±15.9	83.1±16.4
Genetic syndrome*	22 (7.0)	22 (6.3)	12 (4.9)
Aortic valve morphology			
Bicuspid aortic valve	12 (3.8)	24 (7.0)	203 (50.2)
Tricuspid aortic valve	301 (96.2)	321 (93.0)	201 (49.8)
Hypertension	164 (52.1)	181 (52.2)	243 (50.6)
Diabetes mellitus	14 (4.4)	8 (2.3)	32 (6.7)
Hyperlipidaemia	29 (9.2)	35 (10.1)	96 (20.0)
Smoking	113 (39.8)	116 (36.8)	157 (33.2)
Peripheral arterial disease	34 (10.8)	27 (7.8)	67 (22.7)
COPD	30 (9.5)	25 (7.2)	25 (8.5)
Creatinine	82.6±38.0	84.6±37.4	88.1±25.2
Aortic diameter	35.4±7.1	53.3±8.2	52.2±6.6

Data are presented as n (%) or mean±SD.

* Marfan syndrome, Ehlers-Danlos syndrome and Loeys-Dietz syndrome.

ATAAD, acute type A aortic dissection; COPD, chronic obstructive pulmonary disease; TAA, thoracic aortic aneurysm.

## Discussion

The aim of this study was to compare the preoperative cardiovascular risk factors between patients with an ATAAD and those with a TAA. Our findings indicate that hypertension and diabetes mellitus are similarly prevalent in both groups. Hyperlipidaemia, bicuspid aortic valve (BAV) and peripheral arterial disease are less common in patients with ATAAD, while smoking is more frequent among them.

The high proportion of males in both groups aligns with existing literature, which consistently shows a male predominance in both ATAAD and TAA.^22^,^24^ This may be partially attributed to the higher incidence of hypertension in men, a known risk factor for both conditions.^1324^,^26^ However, it is noteworthy that approximately 50% of patients in both groups do not have a history of hypertension, underscoring the complexity of these conditions.

Our study results revealed that hyperlipidaemia is not very common in patients with thoracic aortopathy, with a significantly lower prevalence in patients with ATAAD compared with patients with TAA. Although correlations between the clinical outcomes from this study and the histopathological origin cannot be made, previous studies have shown a lower burden of atherosclerosis both clinically and histopathologically in patients with aortopathy as compared with controls.^13 27^ Lack of atherosclerosis has been associated with a phenotypic switch defect of vascular smooth muscle cells.^28 29^ In the adult healthy aorta, the vascular smooth muscle cells are quiescent and regulate the vascular tone as their primary contractile function. During their lifetime, the vascular smooth muscle cells do not lose their plasticity and can undergo a phenotypic switch from a contractile to a so-called synthetic phenotype, which is distinguished by elevated synthetic and proliferative activity, downregulation of contractile proteins and increased expression and secretion of matrix degrading enzymes.^30^ Patients with thoracic aortopathy are characterised by a phenotypic switch defect of the vascular smooth muscle cells, resulting in a deficiency of contractile vascular smooth muscle cells and an abundance of synthetic cells. Phenotypic switch of vascular smooth muscle cells is crucial for the process of atherogenesis and plaque stability. Therefore, the loss of plasticity and lack of contractile markers in patients with aortopathy might ‘protect’ against the development of atherosclerotic plaques.^13 29^

Peripheral arterial disease is significantly less prevalent in patients with ATAAD compared with patients with TAA. Hyperlipidaemia is a major risk factor for peripheral arterial disease.^31^ Since hyperlipidaemia is less common in patients with ATAAD compared with patients with TAA, this could explain the lower occurrence of peripheral arterial disease among patients with ATAAD. However, the underlying reasons for this discrepancy warrant further investigation.

Smoking, which is a risk factor for both ATAAD and TAA,^24 26^ is more prevalent in patients with ATAAD compared with patients with TAA. In addition, the prevalence of smoking in patients with ATAAD might be under-reported due to the emergency context of ATAAD admissions, unlike patients with TAA who undergo elective surgery with thorough preoperative assessments. However, in the Netherlands, general practitioners continue to play an important role in cardiovascular risk management. They actively screen all patients, from the age of 40, in accordance with their national guidelines.^32^ This proactive approach contributes to earlier detection and treatment of cardiovascular risk factors such as smoking and may support smoking cessation.

The debate concerning the aortic diameter threshold for elective aortic surgery is ongoing.^14^,^17^ Current guidelines recommend aortic replacement surgery at a diameter of 55 mm and above.^18 21^ However, our findings reveal that 83.5% of patients with ATAAD have an aortic diameter below 55 mm, suggesting that many dissections occur below this geometric threshold. Our findings thus raise concerns about the applicability of diameter-based surgical criteria and necessitate the need for a risk stratification model that can aid in patient selection based on more variables than just the geometric threshold.^33^ This model could subsequently assist healthcare professionals in identifying critical risk factors associated with dissection risk, facilitating more personalised patient monitoring and tailored therapeutic interventions.

Diabetes mellitus is infrequent in both patients with ATAAD and patients with TAA, with a particularly low prevalence in ATAAD. Moreover, a lower prevalence of diabetes is observed in patients with dilated ATAAD compared with patients with non-dilated ATAAD. Besides, a lower prevalence of diabetes has previously been extensively described in abdominal aortic pathology,^34 35^ and this was also observed in our recent study in patients with TAA.^27^ Patients with diabetes typically show slower aortic expansion, which might protect against aneurysm formation and an acute aortic dissection.^36^ Although the absolute rate of diabetes is low in both patients with ATAAD and patients with TAA, these rates were not compared with the general population in this study. Our previous study reported a prevalence of diabetes of 13.1% in the general population,^27^ which is relatively higher than observed in patients with aortopathy.

Only a small percentage of patients with ATAAD have a BAV, compared with patients with TAA. Our results are thus in line with the current literature which suggests that bicuspidy does not enhance the risk to develop an acute aortic dissection and a lower threshold for aortic surgery might not be indicated in the guidelines.^37^,^40^

In addition, our findings show that patient height was significantly associated with the occurrence of ATAAD. This observation is in line with current guideline recommendations, which advise indexing aortic diameter to height rather than weight, since height provides a more stable and consistent predictor of aortic risk, whereas weight can fluctuate over time.^18 21^

In conclusion, hypertension and diabetes mellitus are similarly prevalent in both groups. Hyperlipidaemia and peripheral arterial disease are less frequent in patients with ATAAD, whereas smoking is more common. These findings highlight the diversified spread of cardiovascular risk factors between the two conditions and underscore the relevance of targeted preventive approaches. Besides clinical studies, genetic and molecular biological studies are further warranted to develop personalised risk stratification tools.

### Limitations

This study is susceptible to selection and survival bias due to the retrospective study design and the unpredictable nature of ATAAD. Ideally, a prospective study design would be preferable. However, identifying patients at risk of developing an acute aortic dissection remains challenging, which currently limits the feasibility of such prospective studies. Furthermore, only patients who are surgically operated are included. This excludes patients with TAA who did not undergo surgery and are treated conservatively, and patients with ATAAD who did not make it to the hospital or the operating room. Additionally, complete data on cardiovascular risk factors and aortic diameter measurements were not always available due to the emergent presentation of patients, often without prior documented histories or cardiovascular risk assessments. In cases where a CT scan for aortic diameter measurement was not available, transthoracic or transoesophageal ultrasound images were used as an alternative.

## Supplementary material

10.1136/bmjopen-2024-097306 online supplemental file 1

## Data Availability

Data are available upon reasonable request.
