# Supplementary material for: Comparison of cardiovascular risk profiles of patients with type A aortic dissection and thoracic aortic aneurysm: a retrospective multicentre study
Source: BMJ Open. 2025 Sep 23;15(9):e097306. doi: 10.1136/bmjopen-2024-097306 (PMC12458767; doi:10.1136/bmjopen-2024-097306)
Supplement: online supplemental file 1 [file bmjopen-15-9-s001.pdf]

Supplemental table 1: Univariable analysis of ATAAD patients with a diameter <45 mm compared to TAA patients, or ATAAD patients with a diameter ≥45 mm versus TAA patients

	OR	*95% CI*	*P-value*
Aortic diameter <45 mm			
Age	0.988	0.976-1.000	**0.047**
Diabetes mellitus	0.651	0.342-1.241	0.192
Aortic diameter ≥45 mm			
Age	0.996	0.985-1.008	0.518
Diabetes mellitus	0.330	0.150-0.726	**0.006**
