# Supplementary material for: Comparison of cardiovascular risk profiles of patients with type A aortic dissection and thoracic aortic aneurysm: a retrospective multicentre study
Source: BMJ Open. 2025 Sep 23;15(9):e097306. doi: 10.1136/bmjopen-2024-097306 (PMC12458767; doi:10.1136/bmjopen-2024-097306)
Supplement: Supplementary file 2 [file bmjopen-2024-097306.reviewer_comments.pdf]

PEER REVIEW HISTORY

BMJ Open publishes all reviews undertaken for accepted manuscripts. Reviewers are asked to complete a checklist review form and are provided with free text boxes to elaborate on their assessment. These free text comments are reproduced below.

This paper was submitted to another journal from BMJ but declined for publication following peer review. The authors addressed the reviewers' comments and submitted the revised paper to BMJ Open. The paper was subsequently accepted for publication at BMJ Open.

ARTICLE DETAILS

Title (Provisional)

A comparison of cardiovascular risk profiles of patients with type A aortic dissection and thoracic aortic aneurysm: a retrospective multicenter study

Authors

Tirpan, Aytug U; Dolmaci, Onur Baris; Jansen, Evert K; Twisk, Jos W.R.; Klautz, Robert JM; Grewal, Nimrat

---

VERSION 1 - REVIEW

**Reviewer**	**1**
**Name**	**Reviewer 1**
**Affiliation**	
**Date**	**25-Oct-2024**
**COI**	**None**

Comments not published pending reviewer's consent.

VERSION 2 - REVIEW

**Reviewer**	**2**
**Name**	**Howard, Dominic**
**Affiliation**	**University of Oxford, Nuffield Department of Clinical Neurology**
**Date**	**28-Dec-2024**
**COI**	**None**

This study aims to compare the cardiovascular risk profiles of patients diagnosed with Acute Type A Aortic Dissection and those diagnosed with Thoracic Aortic Aneurysms without co-existing aortic dissection. It is a multicentre, retrospective design using patient records from two academic hospitals in the Netherlands, encompassing a large cohort of patients. The study investigates cardiovascular risk factor prevalence in both groups and explores differences, aiming to better understand the pathophysiology of these conditions and potential implications for treatment and risk stratification. The findings contribute to ongoing discussions about aortic disease management, particularly concerning the limitations of aortic diameter as the primary determinant for elective surgery for thoracic aortic aneurysms.

The manuscript appears to be a revised de-novo submission and has been appropriately modified and improved from the original submission. There are however some fundamental weaknesses in the study that need to be addressed:

1. The retrospective study design including only patients receiving intervention is a fundamental flaw and arguably the wrong study design to answer this important epidemiological question. To assess cardiovascular risk profiling for thoracic aortic pathology, a prospective population-based study design would be required including all patients diagnosed with these conditions with access to pre-morbid primary care medical data. Previous prospective population-based studies have documented cardiovascular risk profiles for these pathologies and have been able to investigate risk factor prevalence and control prior to diagnosis/event. In fact, patients with ATAAD who do not survive beyond hospital admission, have been shown to have the highest rates of treatment-resistant hypertension and are more often of female gender. This study would be greatly strengthened if pre-morbid primary care risk factor data and control could have been accessed, including years of smoking, and types (and number) of antihypertensive therapies given prior to event/intervention.

2. Although the study itself is a relatively basic retrospective collection of cardiovascular risk factors from electronic patient records, there are large sections of the introduction and discussion dedicated to the histopathology of thoracic aortic disease, citing the authors previous work. As this study does not include histopathological findings, no direct correlation can be made with the clinical risk profiling identified in this study, and so this has led the authors to interesting, but hypothesis generating, discussion. Although interesting, this is not robust and does not progress our knowledge of these conditions.

3. Overall, although the study is well written and thought provoking, it does not greatly move forward our understanding of these conditions or provide practical improvement in treatment strategy. It would appear that size threshold will continue to be the key indicator for TAA surgery, and there remains no identifier for prediction of a ATAAD event, but better pre-morbid control of modifiable cardiovascular risk factors will potentially reduce future incidence at a population level.

4. Missing data is an issue with the study, as highlighted by the authors, particularly for smoking history and aortic diameter measurements. but there is no information on how missing data was handled. Was any imputation performed, or was a complete case analysis used or were sensitivity analyses performed?

5. Although ATAAD was somewhat defined in the introduction there is no detailed definition of ATAAD or TAA in the methods section. These need to be added. For instance, were TAAs only ascending aortic TAAs or any type of thoraco-abdominal aneurysm. Were false aneurysms included? Were TAAs arising from old aortic dissections included?

**Reviewer**	**3**
**Name**	**Van Der Linden, Jan**
**Affiliation**	**Karolinska Institute**
**Date**	**02-Jun-2025**
**COI**	**None**

This is an interesting and well written study. I have the following comments that may help to improve the manuscript.

The first three points concern possible bias that may severely limit the conclusions of the paper. Please answer these questions and include your answers in the manuscript.

1. What percentage of the ATAAD patients did not have a regular contact with a GP or a clinic for their aortic disease before the operation? Would that also possibly result in a lacking hyperlipidemia condition in the patients´ medical history? Could that also have influenced, i.e. decreased, the reported percentage of lipid-lowering medication and cholesterol values?

2. How did you diagnose the occurrence of peripheral disease in both categories of patients? This may be especially interesting in ATAAD patients as it may underestimate occurrence of peripheral disease if some of these patients did not have a history of regular contact with a clinic.

3. How was smoking diagnosed in the two categories of patients and could a non-regular contact with a GP/clinic in ATAAD patients have influenced the results (lack of support to quit smoking)?

4. Why did you adjust for age when the mean difference was minimal and not significantly different between the groups? Please include an explanation in the manuscript.

5. Please state the used indications for elective operations of TAA in the study as 61% of the patients had a diameter of the ascending aorta of less than 55 mm considering that in current European and American guidelines, the threshold for aortic replacement surgery is 55 mm and greater.

VERSION 2 - AUTHOR RESPONSE

REVIEWER 2
Dominic Howard, University of Oxford

Comments to the Author:
This study aims to compare the cardiovascular risk profiles of patients diagnosed with Acute Type A Aortic Dissection and those diagnosed with Thoracic Aortic Aneurysms without co-existing aortic dissection. It is a multicentre, retrospective design using patient records from two academic hospitals in the Netherlands, encompassing a large cohort of patients. The study investigates cardiovascular risk factor prevalence in both groups and explores differences, aiming to better understand the pathophysiology of these conditions and potential implications for treatment and risk stratification. The findings contribute to ongoing discussions about aortic disease management, particularly concerning the limitations of aortic diameter as the primary determinant for elective surgery for thoracic aortic aneurysms.

The manuscript appears to be a revised de-novo submission and has been appropriately modified and improved from the original submission. There are however some fundamental weaknesses in the study that need to be addressed:

1. The retrospective study design including only patients receiving intervention is a fundamental flaw and arguably the wrong study design to answer this important epidemiological question. To assess cardiovascular risk profiling for thoracic aortic pathology, a prospective population-based study design would be required including all patients diagnosed with these conditions with access to pre-morbid primary care medical data. Previous prospective population-based studies have documented cardiovascular risk profiles for these pathologies and have been able to investigate risk factor prevalence and control prior to diagnosis/event. In fact, patients with ATAAD who do not survive beyond hospital admission, have been shown to have the highest rates of treatment-resistant hypertension and are more often of female gender. This study would be greatly strengthened if pre-morbid primary care risk factor data and control could have been accessed, including years of smoking, and types (and number) of antihypertensive therapies given prior to event/intervention.

Thank you for your valuable comment. We acknowledge that our study includes only patients who received an intervention, which may introduce selection bias. Ideally, a prospective, population-based study design would be the most appropriate to fully capture the cardiovascular risk profile in these patients. However, this is particularly challenging in the context of acute type A aortic dissections, as it is not yet possible to prospectively identify which patients will eventually develop an aortic dissection. Only studying patients with a thoracic aortic aneurysm is in our opinion also not sufficient for this purpose since previous studies have shown a relatively large portion of patients that develop an aortic dissection without a preceding aortic aneurysm (1).

Our current retrospective study represents a first step and highlights potential clinical associations that can inform the design of future prospective studies. We aim to develop a more comprehensive, population-based study in the future.

We furthermore believe that the risk for selection bias is reduced due to the well-developed emergency transport system in the Netherlands. This system in combination with a small country (the Netherlands) and the high density of hospitals results in short median transport times (usually within 15 minutes) results in a lower survival bias. This is also seen in our cohort that includes patients that arrived alive but died before or during surgery. While we acknowledge that this does not eliminate the possibility of bias entirely, we believe its impact on our findings is limited.
Lastly, we agree that the pre-morbid data of antihypertensive therapies as well as the number of smoking years is missing and could have given a more in-depth evaluation of the cardiovascular risk factors in our cohort. However, given the acute and unpredictable presentation of ATAAD, such information is often unavailable due to a lack of documentation at hospital admission due to the emergent setting.

We have made the following changes in our limitation on page 17, line 2-6:

This study is susceptible to selection and survival bias due to the retrospective study design and the acute unpredictable nature of type A aortic dissections. Ideally, a prospective study design would be preferable. However, identifying patients at risk for developing an acute aortic dissection remains challenging, which currently limits feasibility of such prospective studies.

And on page 17, line 8-12:
Additionally, complete data on cardiovascular risk factors and aortic diameter measurements were not always available due to the emergent presentation of patients, often without prior documented histories or cardiovascular risk assessments.

2. Although the study itself is a relatively basic retrospective collection of cardiovascular risk factors from electronic patient records, there are large sections of the introduction and discussion dedicated to the histopathology of thoracic aortic disease, citing the authors previous work. As this study does not include histopathological findings, no direct correlation can be made with the clinical risk profiling identified in this study, and so this has led the authors to interesting, but hypothesis generating, discussion. Although interesting, this is not robust and does not progress our knowledge of these conditions.

Thank you for your comment. We agree that our study does not include histopathological data and therefore cannot establish a direct correlation between histological findings and clinical risk profiles. The sections on histopathology of aortopathy in the introduction and discussion were intended to provide context and illustrate the complexity and multifactorial nature of aortic disease. We believe this provides valuable insights into a relatively rare and challenging patient population, particularly given the acute and often unpredictable presentation of aortic dissection. We have added the following sentence in the discussion to clarify that no direct correlation can be made on page 14, line 15-18:

Although correlations between the clinical outcomes from this study and the histopathological origin cannot be made, previous studies have shown a lower burden of atherosclerosis both clinically and histopathologically in aortopathy patients as compared to controls (13, 29).

3. Overall, although the study is well written and thought provoking, it does not greatly move forward our understanding of these conditions or provide practical improvement in treatment strategy. It would appear that size threshold will continue to be the key indicator for TAA surgery, and there remains no identifier for prediction of a ATAAD event, but better pre-morbid control of modifiable cardiovascular risk factors will potentially reduce future incidence at a population level.

Thank you for your comment. We acknowledge that this retrospective analysis alone is insufficient to immediately impact clinical practice. However, by identifying differences in cardiovascular risk profiles between ATAAD and TAA patients, this study provides critical groundwork for future prospective and interventional studies aimed at improving clinical risk assessment beyond the current diameter-based guidelines (1-3). We are actively working towards a prospective approach that integrates additional clinical and biological factors to develop a more precise, individualized risk prediction model, thereby potentially improving clinical decision-making and patient outcomes in the long term.

We appreciate the reviewer's observation and have added the following sentence to the discussion on page 15, line 24 and page 16, line 1-2:

This model could subsequently assist healthcare professionals in identifying critical risk factors associated with dissection risk, facilitating more personalized patient monitoring and tailored therapeutic interventions.

4. Missing data is an issue with the study, as highlighted by the authors, particularly for smoking history and aortic diameter measurements. but there is no information on how missing data was handled. Was any imputation performed, or was a complete case analysis used or were sensitivity analyses performed?

Thank you for highlighting this important issue. We acknowledge that missing data represent a limitation of our study. In this analysis, no imputation methods or sensitivity analysis were performed. Instead, we employed an available-case approach, meaning all patients were included in the analysis even when certain variables contained missing values. This approach allows us to maximize the utilization of available data and retain statistical power. To clarify our method, we have added the following sentence to the method section (page 10, line 4-6):

All available patients were included in the statistical analysis, even if some variables contained missing values, to maximize data utilization and preserve statistical power.

5. Although ATAAD was somewhat defined in the introduction there is no detailed definition of ATAAD or TAA in the methods section. These need to be added. For instance, were TAAs

only ascending aortic TAAs or any type of thoraco-abdominal aneurysm. Were false aneurysms included? Were TAAs arising from old aortic dissections included?

Thank you for your valuable suggestion. We agree that a clear definition of the included ATAAD and TAA cases is essential for reproducibility. In our study, acute type A aortic dissections (ATAAD) were defined as cases requiring surgical replacement of the aortic root, ascending aorta, or aortic arch due to an acute dissection. Thoracic aortic aneurysms (TAA) were defined as cases with aortic diameters ≥45 mm requiring surgical intervention in the absence of dissection. We excluded thoraco-abdominal aneurysms, TAAs arising from previous dissections, and other rare conditions such as unicuspid or quadricuspid aortic valves to maintain a homogeneous study population. Thoracic false aneurysms were included only when associated with a spontaneous ATAAD, excluding cases due to trauma or iatrogenic causes.

To clarify this, we have added and amended the following sentences in the method section on page 7, line 6-9:

Patients who required surgical replacement of the aortic root, ascending aorta or aortic arch due to an aortic dissection were included in the ATAAD group. Patients with an aortic diameter of ≥45mm without evidence of dissection were included in the TAA group.

And on page 7, line 17-24:
To ensure a targeted and homogeneous study population, thoraco-abdominal aneurysms were excluded from the analysis. Thoracic false aneurysms were included only when associated with a spontaneous ATAAD, excluding cases due to trauma or iatrogenic causes. In addition, TAA's arising from chronic dissections, and cases with unicuspid and quadricuspid aortic valves were excluded due to their rarity.

REVIEWER 3
Jan Van Der Linden, Karolinska Institute

Comments to the Author:
This is an interesting and well written study. I have the following comments that may help to improve the manuscript.
The first three points concern possible bias that may severely limit the conclusions of the paper. Please answer these questions and include your answers in the manuscript.

1. What percentage of the ATAAD patients did not have a regular contact with a GP or a clinic for their aortic disease before the operation? Would that also possibly result in a lacking hyperlipidemia condition in the patients´ medical history? Could that also have influenced, i.e. decreased, the reported percentage of lipid-lowering medication and cholesterol values?

Thank you for this insightful question. In our cohort, the majority of patients were under regular follow-up at cardiology clinics in accordance with ESC guidelines (3), with follow-up intervals typically ranging from every 6 months to once every 3 years. Consequently, data on lipid levels and the use of lipid-lowering medication use were generally well-documented.

However, we acknowledge that a small subset of patients presenting with acute aortic dissection had no prior contact with healthcare services. For these patients, data on cardiovascular risk factors, including lipid levels and medications use, may have been unavailable. While these cases were limited, they may have contributed to underreporting in this domain.

To clarify the point, we have added the following sentence to the limitation section on page 17, line 8-12:

Additionally, complete data on cardiovascular risk factors and aortic diameter measurements were not always available due to the emergent presentation of patients, often without prior documented histories or cardiovascular risk assessments.

2. How did you diagnose the occurrence of peripheral disease in both categories of patients? This may be especially interesting in ATAAD patients as it may underestimate occurrence of peripheral disease if some of these patients did not have a history of regular contact with a clinic.

Thank you for this valuable question. Peripheral arterial disease (PAD) was identified based on documentation in the patient's medical history. In the Netherlands, PAD is predominantly managed by general practitioners and vascular surgeons, with diagnosis and treatment generally adhering to the European Society of Cardiology guidelines (3). According to these guidelines, PAD is diagnosed based on a resting ankle-brachial index ≤ 0.90. In cases where the ankle-brachial index was > 0.90, a >20% post-exercise decrease in ankle-brachial index and/ or confirmatory imaging (e.g. duplex ultrasound or angiography) was considered diagnostic and documented according to the treating physician.

To clarify this in the manuscript, we have added the following sentence in the method section to clarify this (page 9, line 12-14):

Peripheral arterial disease was scored if documented in the patient's medical history, which is based on ESC guidelines: a resting ankle-brachial index ≤0.90. If >0.90, a >20% post-exercise decrease and/or supportive imaging findings also support the diagnosis (19).

We acknowledge that the prevalence of peripheral arterial disease could be underestimated in the ATAAD group due to the acute presentation and the possibility that some patients had no prior diagnosis or regular medical follow-up prior to the event. We have amended the following sentence in the limitations, to clarify this point (page 17, line 8-12):

Additionally, complete data on cardiovascular risk factors and aortic diameter measurements were not always available due to the emergent presentation of patients, often without prior documented histories or cardiovascular risk assessments.

3. How was smoking diagnosed in the two categories of patients and could a non-regular contact with a GP/clinic in ATAAD patients have influenced the results (lack of support to quit smoking)?

Thank you for your question. Smoking status was assessed based on patient self-report

during clinical intake or follow-up. Patients were classified as smokers if they were current smokers or had a history of smoking. To clarify this, we have added the following sentence to the method section (page 9, line 11-12):

Patients were scored as smokers if they had ever smoked in the past or were current smokers.

We acknowledge that patients with ATAAD may have had less consistent contact with a general practitioner prior to their acute presentation, potentially limiting access to preventive measures such as smoking cessation support. However, in the Netherlands, general practitioners play a central role in cardiovascular risk management and routinely screen individuals at the age of 40 years, in line with national guidelines (4). This proactive approach contributes to an earlier identification and management of cardiovascular risk factors such as smoking, even among patients without a known cardiovascular condition. As such, it may partially compensate for the absence of cardiology follow-up in some patient who later develop ATAAD.
We have added the following sentences in the discussion to clarify this (page 15, line 13-17):
However, in the Netherlands, general practitioners continue to play an important role in cardiovascular risk management. They actively screen all patients, from the age of 40, in accordance with their national guidelines (34). This proactive approach contributes to earlier detection and treatment of cardiovascular risk factors such as smoking and may support smoking cessation.

4. Why did you adjust for age when the mean difference was minimal and not significantly different between the groups? Please include an explanation in the manuscript.

Thank you for this important point. Although the mean age did not differ significantly between the groups, age remains a well-established clinical risk factor in aortic disease and may influence cardiovascular risk profiles. Even small differences in age can have meaningful effects in outcomes. Therefore, we adjusted for age as a covariate to control for potential confounding and to enhance the robustness and generalizability of our findings.
We have added the following explanation to the methods section, on page 10, line 8-10:

Although mean age did not differ statistically between groups, it was included as a covariate in the analysis because of its known clinical relevance in aortic disease. This was to account for potential confounding effects and to improve generalizability.

5. Please state the used indications for elective operations of TAA in the study as 61% of the patients had a diameter of the ascending aorta of less than 55 mm considering that in current European and American guidelines, the threshold for aortic replacement surgery is 55 mm and greater.

Thank you for your comment. Surgical decisions were made in accordance with the ESC and STS/EACTS guidelines (2, 3). Although the general threshold for elective surgery for TAA is ≥55 mm, the guidelines also state that surgery with smaller diameters may be considered in specific situations. Analogous to the ESC and STS/EACTS guidelines, surgery below 55 mm was performed in patients with connective tissue disorders (e.g. Marfan syndrome), risk

factors (e.g. hypertension, aortic growth rate >3 mm/year, or family history), or the need for concomitant aortic valve surgery, which according to current guidelines warrants surgery at ≥50 mm for tricuspid valves and ≥45 mm for bicuspid valves. Importantly, all surgical decisions in our study were made by multidisciplinary heart teams, ensuring strict adherence to guideline-based and individualized clinical decision-making.

We have clarified this in the methods section (page 7, lines 9–16):
Surgical decisions were made in accordance with the ESC and STS/EACTS guidelines (18, 19). Although the general threshold for elective surgery for TAA is ≥55 mm, the guidelines also state that surgery with smaller diameters may be considered in specific situations. Analogous to the ESC and STS/EACTS guidelines, surgery below 55 mm was performed in patients with connective tissue disorders (e.g. Marfan syndrome), risk factors (e.g. hypertension, aortic growth rate >3 mm/year, or family history), or concomitant aortic valve/root pathology. All surgical indications were determined by multidisciplinary heart teams, ensuring guideline-based decision-making.

References

1. Pape LA, Tsai TT, Isselbacher EM, Oh JK, O'Gara P T, Evangelista A, et al. Aortic diameter >or = 5.5 cm is not a good predictor of type A aortic dissection: observations from the International Registry of Acute Aortic Dissection (IRAD). Circulation. 2007;116(10):1120-7.
2. Czerny M, Grabenwöger M, Berger T, Aboyans V, Della Corte A, Chen EP, et al. EACTS/STS Guidelines for diagnosing and treating acute and chronic syndromes of the aortic organ. Eur J Cardiothorac Surg. 2024;65(2).
3. Mazzolai L, Teixido-Tura G, Lanzi S, Boc V, Bossone E, Brodmann M, et al. 2024 ESC Guidelines for the management of peripheral arterial and aortic diseases. Eur Heart J. 2024;45(36):3538-700.
4. Nederlands Huisartsen Genootschap. NHG-Standaard Cardiovasculair risicomanagement 2019 [updated 2024. Available from: https://richtlijnen.nhg.org/standaarden/cardiovasculair-risicomanagement.

VERSION 3 - REVIEW

Reviewer **2**

Name **Howard, Dominic**

Affiliation **University of Oxford, Nuffield Department of Clinical Neurology**

Date **09-Jul-2025**

COI

The authors have revised the manuscript appropriately and it is significantly improved.

**Reviewer**	**3**
**Name**	**Van Der Linden, Jan**
**Affiliation**	**Karolinska Institute**
**Date**	**09-Jul-2025**
**COI**	

Thank you very much for the revision. The manuscript has improved considerably. However, I am still a bit concerned about the impact of missing values. I would like you to present number of missing data for each variable in the tables. Also, you write "All available patients were included in the statistical analysis, even if some variables contained missing values, to maximize data utilization and preserve statistical power." Contrary to your statement, as I understand it, missing values for each variable in a multivariable will decrease statistical power especially if a lot of data are missing for a few variables. Thus, I suggest a specialist statistical review.

VERSION 3 - AUTHOR RESPONSE

REVIEWER 2
Dominic Howard, University of Oxford

Comments to the Author:
The authors have revised the manuscript appropriately and it is significantly improved.

We thank the reviewer for their encouraging feedback and are pleased that the revisions have significantly improved the manuscript.

REVIEWER 3
Jan Van Der Linden, Karolinska Institute

Comments to the Author:
Thank you very much for the revision. The manuscript has improved considerably. However, I am still a bit concerned about the impact of missing values. I would like you to present number of missing data for each variable in the tables. Also, you write "All available patients were included in the statistical analysis, even if some variables contained missing values, to maximize data utilization and preserve statistical power." Contrary to your statement, as I understand it, missing values for each variable in a multivariable will decrease statistical power especially if a lot of data are missing for a few variables. Thus, I suggest a specialist statistical review.

We thank the reviewer for their constructive feedback and are glad that the manuscript has improved considerably following the revisions. We appreciate the concern regarding missing values and have addressed this issue in the revised manuscript.

The number of missing values for each variable has now been added in Table 1 (on page 20 and 21). Categorical variables are presented as n [n = initially available cases] (%) and continuous variables as mean ± SD [n = initially available cases], where "initially available cases" indicates the number of cases available for analysis.

The reviewer is correct that our previous statement regarding missing values was inaccurate: missing data in a multivariable analysis can indeed reduce statistical power, particularly if substantial data are missing for some variables. We consulted a statistician regarding the analysis, who advised several changes to appropriately handle missing values; this statistician is also included in the author contributions.

To address the missing values, multiple imputation was performed using fully conditional specification (chained equations) with predictive mean matching, generating forty imputed datasets (page 8). Initially, BMI, weight, and height were included in the analysis; however, since these variables are highly correlated, BMI was removed and only weight and height were retained. A univariable analysis was then performed, as illustrated in Table 1 (page 20 and 21). Following this, a multivariable analysis was conducted; the inclusion of aortic diameter, which had previously been limited by missing data, was now possible after imputation (page 22).

The multiple imputation largely confirmed the previous results, although some variables changed in significance. In the new multivariable analysis, sex and height became significant, and aortic diameter could also be included and was significant. On the advice of the statistician, additional confounding by sex and age was removed, as well as interactions as effect modifiers, resulting in clearer and more distinct results.

Consequently, we amended the discussion section to reflect these findings:

Since sex became significant, we updated the discussion with the following sentence on page 13, line 8-10:

The high proportion of males in both groups aligns with existing literature, which consistently shows a male predominance in both ATAAD and TAA [22-24].

Since height became significant, we added the following text on page 15, line 16-19:

In addition, our findings show that patient height was significantly associated with the occurrence of ATAAD. This observation is in line with current guideline recommendations, which advise indexing aortic diameter to height rather than weight, since height provides a more stable and consistent predictor of aortic risk, whereas weight can fluctuate over time [18, 21].

These changes have been incorporated throughout the manuscript to accurately reflect the updated statistical analysis and results.

VERSION 4 - REVIEW

**Reviewer**	**3**
**Name**	**Van Der Linden, Jan**
**Affiliation**	**Karolinska Institute**
**Date**	**06-Sep-2025**
**COI**	

No more comments
